# Beyond Symmetry and Expansion: Coaxiality and Its Impact on Long-Term TAVR Outcomes

**DOI:** 10.1016/j.jacadv.2025.102182

**Published:** 2025-09-17

**Authors:** Ioannis Skalidis, Philippe Garot, Bernard Chevalier, Hakim Benamer, Mariama Akodad

**Affiliations:** Institut Cardiovasculaire Paris-Sud, Hôpital Jacques Cartier, Ramsay-Santé, Massy, France

The recent study by Angellotti et al^1^ provides valuable insights into the clinical impact of transcatheter heart valve (THV) deployment geometry, introducing coaxiality as a novel and independent dimension of procedural optimization. While valve expansion and symmetry have been recognized as critical to favorable transcatheterac aortic valve replacement (TAVR) outcomes, the demonstration that noncoaxial implantation is associated with significantly reduced early device safety and a more than threefold increase in stage-2 bioprosthetic valve failure at 1 year (HR: 3.47; 95% CI: 1.26-9.54) positions coaxiality as a parameter of comparable importance.^2^

Given that younger, lower-risk populations are increasingly receiving TAVR and may eventually require repeat procedures, the need for precise and durable initial deployment is paramount. This study raises an important consideration: should coaxial alignment now be actively targeted as part of the implantation strategy alongside traditional parameters such as THV expansion, commissural alignment, or implant depth? The authors show that higher left ventricular outflow tract calcium and use of self-expanding valves are predictors of noncoaxiality, while predilatation is protective. This observation invites discussion about the potential benefits of systematic predilatation in anatomically complex cases to improve coaxiality, even as its use has declined in many centers.

Moreover, the association between increased axial angle and significantly higher rates of new permanent pacemaker implantation (33.6% in the highest tertile, aHR: 4.49; *P* < 0.001) adds a mechanistic dimension: could asymmetric prosthesis orientation directly alter local mechanical stress on conduction tissues? The underlying interactions deserve further exploration, ideally with 3-dimensional computed tomography imaging modalities that can capture the relationship between the implanted THV and surrounding anatomical structures more comprehensively than fluoroscopy.

This study by Angellotti et al^1^ is an important contribution that expands the TAVR optimization paradigm. Prospective validation, integration of coaxiality into preprocedural planning algorithms, and long-term assessments of valve durability in relation to axial alignment are warranted. As TAVR expands into younger demographics, optimizing every element of deployment—including coaxiality—becomes not only desirable but necessary.
